# SOX17 increases the cisplatin sensitivity of an endometrial cancer cell line

**DOI:** 10.1186/s12935-016-0304-7

**Published:** 2016-04-08

**Authors:** Yongli Zhang, FeiZhou Jiang, Wei Bao, Huilin Zhang, XiaoYing He, Huihui Wang, Xiaoping Wan

**Affiliations:** Department of Obstetrics and Gynecology, Shanghai First People’s Hospital Affiliated to Shanghai Jiao Tong University, No. 650 Xinsongjiang Road, Shanghai, 201620 China; Department of Obstetrics and Gynecology, International Peace Maternity & Child Health Hospital Affiliated to Shanghai Jiao Tong University School of Medicine, No. 910 Hengshan Road, Shanghai, 200031 China; Department of Obstetrics and Gynecology, Shanghai First Maternity and Infant Hospital, Tongji University School of Medicine, No. 2699 Gaokexi Road, Shanghai, 201204 China

**Keywords:** SOX17, Endometrial cancer, Cisplatin, Chemoresistance, Apoptosis

## Abstract

**Background:**

Endometrial cancer (EC) is the most common form of malignant gynecological tumor. Treatment with cisplatin (CDDP) is the mainstay of EC chemotherapy. The apoptotic machinery is regarded as an important etiological factor in chemoresistance. Recent evidence has suggested that overexpression of the transcription factor SOX17 prevented apoptosis in tumor cell lines. The effect of SOX17 on apoptosis in EC cisplatin chemoresistance remains unclear.

**Methods:**

Immunohistochemistry and the reverse transcription-polymerase chain reaction were employed to detect gene expression in paraffin-embedded EC tissues and blood samples. The anti-proliferative ability of SOX17 on EC cells was assessed by MTT. Flow cytometric analysis was used to detect cell apoptosis by annexin V/PI double-staining. The expression of apoptosis-related proteins was analyzed by western blot. In the in vivo study, nude mice were subcutaneously injected with EC cells, and received cisplatin treatment through intraperitoneal chemotherapy. Apoptosis of in vivo samples was analyzed by *TUNEL* assay.

**Results:**

SOX17 expression decreased the chemical resistance of EC cells to CDDP. HEC-1B cells with an elevated expression of SOX17 had a lower cell viability and higher apoptosis rate after cisplatin exposure. Overexpression SOX17 up-regulated wild type p53 after being exposed to cisplatin, while the expression of BCL2-associated X protein and cleaved caspase-3 simultaneously increased. Caspase-9 inhibitor reduced the efficacy of SOX17 in HEC-1B cells after cisplatin treatment. In the in vivo study, SOX17 overexpression clearly restrained the tumor growth and increased the cisplatin toxicity and apoptosis of tumor cells.

**Conclusions:**

SOX17 is involved in the p53-mediated apoptosis pathway, and increases the sensitivity of HEC-1B cells to cisplatin.

**Electronic supplementary material:**

The online version of this article (doi:10.1186/s12935-016-0304-7) contains supplementary material, which is available to authorized users.

## Background

Endometrial cancer (EC) is the most common form of gynecological malignancy, with an estimated 47,130 new cases and 8010 deaths occurring in 2014 [1]. Chemotherapy combined with whole pelvic external beam radiotherapy is the key procedure in improving the survival rate of patients with clinical II/III stage disease [2]. CDDP is the first-line chemotherapeutic agent for EC treatment. The pharmacological action of CDDP is to induce tumor cell apoptosis by creating inter- and intra-strand DNA adducts through irreversible intercalation, thereby inducing both DNA damage and apoptotic responses [3]. CDDP induces apoptosis only in sensitive cells, not in their resistant counterparts [4]. CDDP resistance is a common phenomenon in EC impeding successful cancer treatment, the underlying molecular mechanism of which is not well understood.

The importance of the transcription factor SOX17 (SRY-box containing gene 17) in EC cell biology has recently aroused great interest. SOX17 belongs to the high mobility group (HMG) box transcription factor superfamily, which is homologous to the sex-determining gene SRY [5]. SOX17 has been recognized as an important antagonist and inhibitor of the canonical Wnt signaling pathway [6]. Human SOX17 was found to encode a 414-amino-acid protein containing a HMG box, which has been implicated in embryogenesis [7].

Apoptotic disorders are among the many mechanisms significant in carcinogenesis [8]. An earlier study has shown that SOX17 overexpression prevents apoptosis in oligodendroglioma (HOG) cells [3]. Moreover, the apoptotic machinery has been considered to be an important etiological factor in chemoresistance. The effect of SOX17 on apoptosis in EC chemoresistance has not been investigated.

The TP53 gene, a guardian of cell genome stability, is one of the most extensively researched genes involved in apoptosis. Abnormal p53 protein production leads to an inability to repair damaged DNA as well as uncontrollable cancer cell growth [9–11]. One of the mechanisms underlying CDDP resistance is the inhibition of cell apoptosis through the increase in p53 protein levels.

In the cytosol, p53 induces cell death by forming inhibitory complexes with Bcl-XL and Bcl-2, which leads to the permeabilization of the mitochondrial membrane and the release of cytochrome C [12]. Mutation of p53 has been strongly associated with chemoresistance of lung [13], ovarian [14] and gastric [15] cancers. In breast cancer, tumors containing p53 mutants are enriched for the SOX17 gene [16].

However, the role of SOX17 in EC chemoresistance is not completely understood. Based on these findings, we investigated whether SOX17 regulates chemosensitivity in EC. Here we found that up-regulation of SOX17 improved p53 expression, which in turn sensitized EC cells to CDDP.

## Results

### SOX17 expression is decreased in chemo-resistant endometrial cancer

**Fig. 1:**
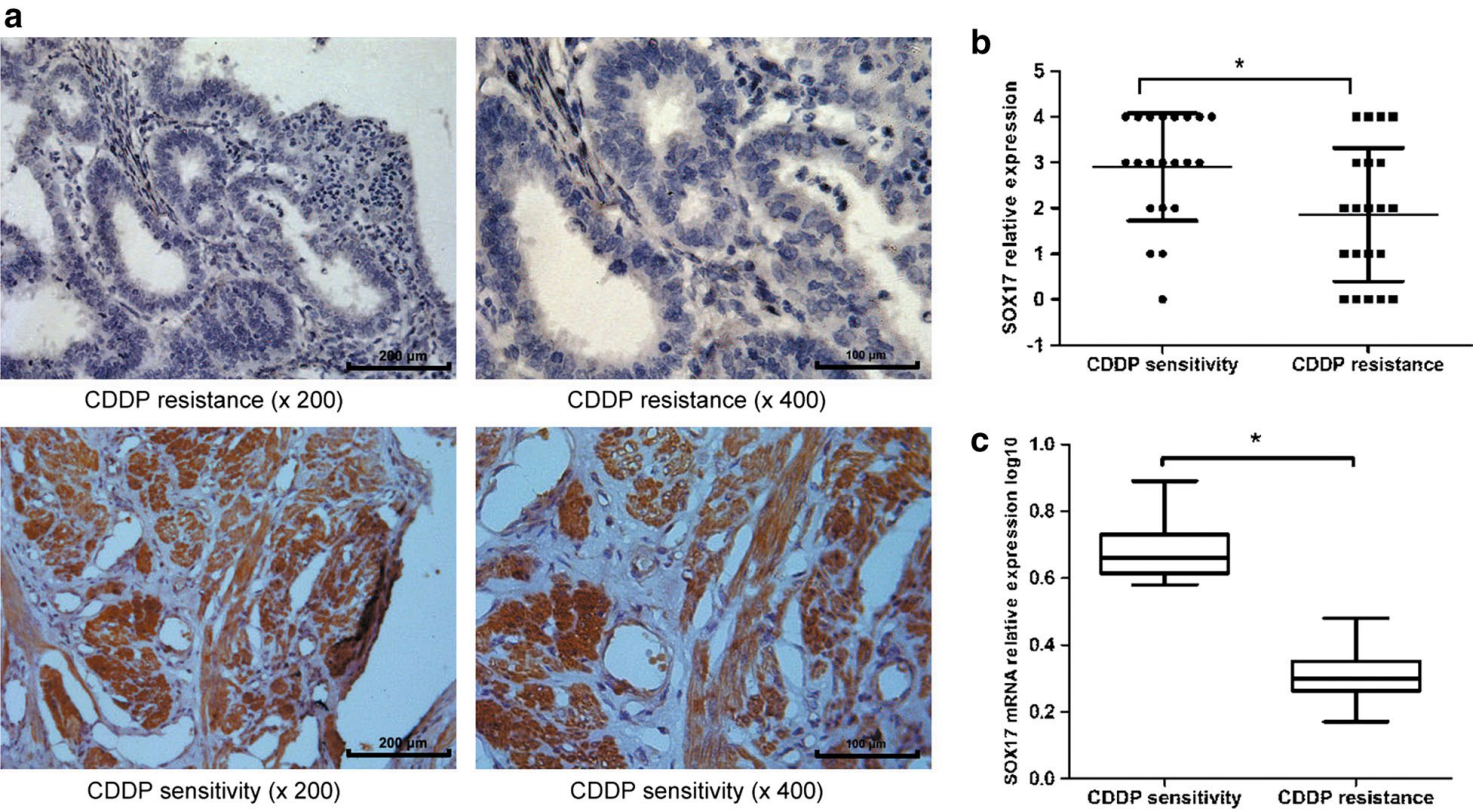
SOX17 down-regulated expression in chemo-resistant endometrial cancer. **a**, **b** IHC analysis of the expression of SOX17 in EC tissues. The levels of SOX17 proteins are higher in chemo-sensitive EC tissues. Original magnification: ×200 (*left*), ×400 (*right*). **c** The analyses of the SOX17 expression levels were performed on blood samples from cisplatin-sensitive patients (n = 17) and cisplatin-resistant patients (n = 12). All data were expressed as fold change relative to the expression of GAPDH in the tissue (control, expression = 1). The results were expressed as Log 10 (2^−△△Ct^) (*P < 0.05)

Patients with EC who have received comprehensive staging surgery and suffered disease relapse within 6 months after regular platinum containing therapy are considered to be platinum resistant [17]. We selected a group of 21 chemo-resistant and another of 21 chemo-sensitive patients. To confirm the expression levels of SOX17, we assayed SOX17 expression in paraffin-embedded tissues obtained from all 42 EC patients. SOX17 levels were lower in the chemo-resistant tissues (Fig. 1a, b; Additional file 1: Table S1). There were no significant differences between the clinical features of the patients of the two groups (Additional file 1: Table S2). To clarify the molecular mechanisms underlying CDDP resistance in EC cells, SOX17 expression levels in 29 EC patients were assayed after cisplatin-based combination chemotherapy. The results showed that SOX17 expression in blood samples from chemo-resistant patients was significantly decreased compared with that from chemo-sensitive patients (Fig. 1c).

### Generation and identification of transiently transfected cells

**Fig. 2:**
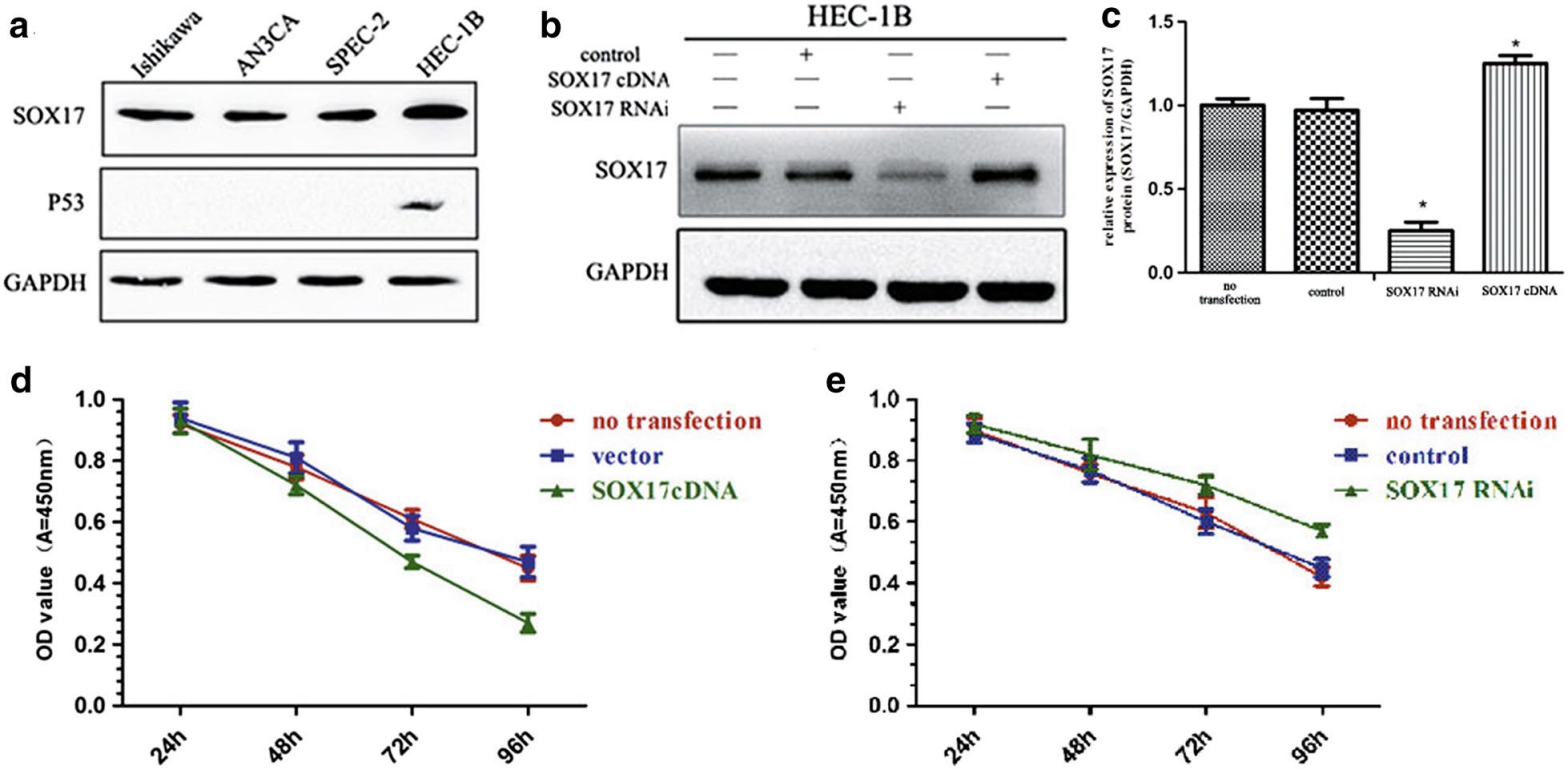
Effects of SOX17 on the chemosensitivity of HEC-1B cells to CDDP. **a** The expression level of SOX17 in various EC cells as determined by western blot. **b** SOX17 expression level in HEC-1B cells at 72 h post transfection. **c** The density quantification of the western blot bands from **b**. **d** The proliferation of SOX17 cDNA transfected cells at different time points post CDDP treatment. CDDP sensitivity was increased in cells transfected with SOX17 cDNA. **e** Decreased CDDP sensitivity was found in SOX17 shRNA transfected cells (*P < 0.05)

Western blot analyses were performed to assay the expression of SOX17 in the EC cell lines. SOX17 protein was expressed in all the EC cell lines (Fig. 2a). However, only HEC-1B cells expressed high levels of p53 protein under basal conditions. To further investigate the function of SOX17 in EC cells, SOX17 overexpression and SOX17 knockdown plasmids were constructed and were used to transfect HEC-1B cells. The cells were used for further study at 72 h post transfection, and the expression levels of SOX17 were confirmed using western blot analysis (Fig. 2b, c).

### SOX17 increased the sensitivity of HEC-1B cells to CDDP

Cytotoxicity tests were performed to study the influence of SOX17 on the cytotoxicity of CDDP to HEC-1B cells. After transfected for 72 h, the cells were treated with CDDP (20 μg/ml) for 24, 48, 72, 96 h. Cells overexpressing SOX17 displayed a higher sensitivity to CDDP and a lower cell viability than the control (Fig. 2d), whereas cells with a reduced expression of SOX17 had a lower sensitivity to CDDP and an enhanced cell viability (Fig. 2e). These data suggested that SOX17 increased the CDDP sensitivity of EC cells.

**Fig. 3:**
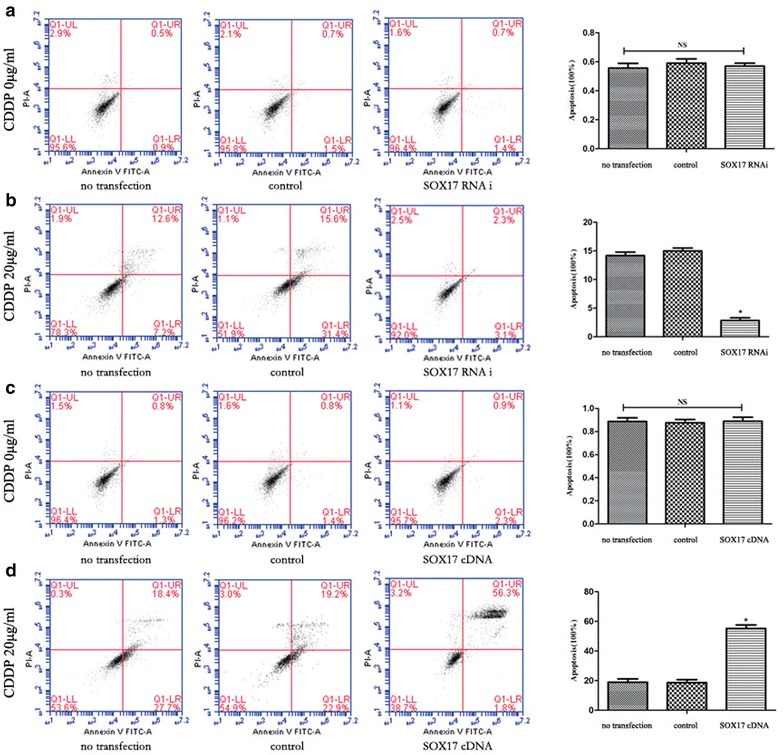
Apoptosis level at 48 h after CDDP treatment in HEC-1B cells transfected with either SOX17 shRNA or SOX17 cDNA. **a** The apoptosis level of cells 120 h post SOX17 shRNA transfection. **b** The apoptosis level of SOX17 shRNA transfected cells 48 h post CDDP treatment. **c** The apoptosis level of cells 120 h post SOX17 cDNA transfection. **d** The apoptosis level 48 h post CDDP treatment in cells transfected with SOX17 cDNA (*P < 0.05)

To further assay the effect of SOX17 on the sensitivity of HEC-1B cells to CDDP, we used flow cytometry to examine the apoptosis rate of HEC-1B cells after treatment with CDDP (20 μg/ml) for 48 h. The apoptosis rate of HEC-1B cells over-expressing SOX17 was higher than that of the control group (*P* < 0.05). Meanwhile, under-expression of SOX17 decreased the apoptosis rate (*P* < 0.05). The apoptosis rate after treatment with CDDP was 55.1 ± 2.47 and 2.36 ± 0.47 % in SOX17 up-regulated and down-regulated groups, respectively (*P* < 0.05) (Fig. 3a–d). These results further suggested that SOX17 increased the CDDP sensitivity of HEC-1B cells.

### SOX17 overexpression sensitizes EC cell to cisplatin by increasing apoptosis

**Fig. 4:**
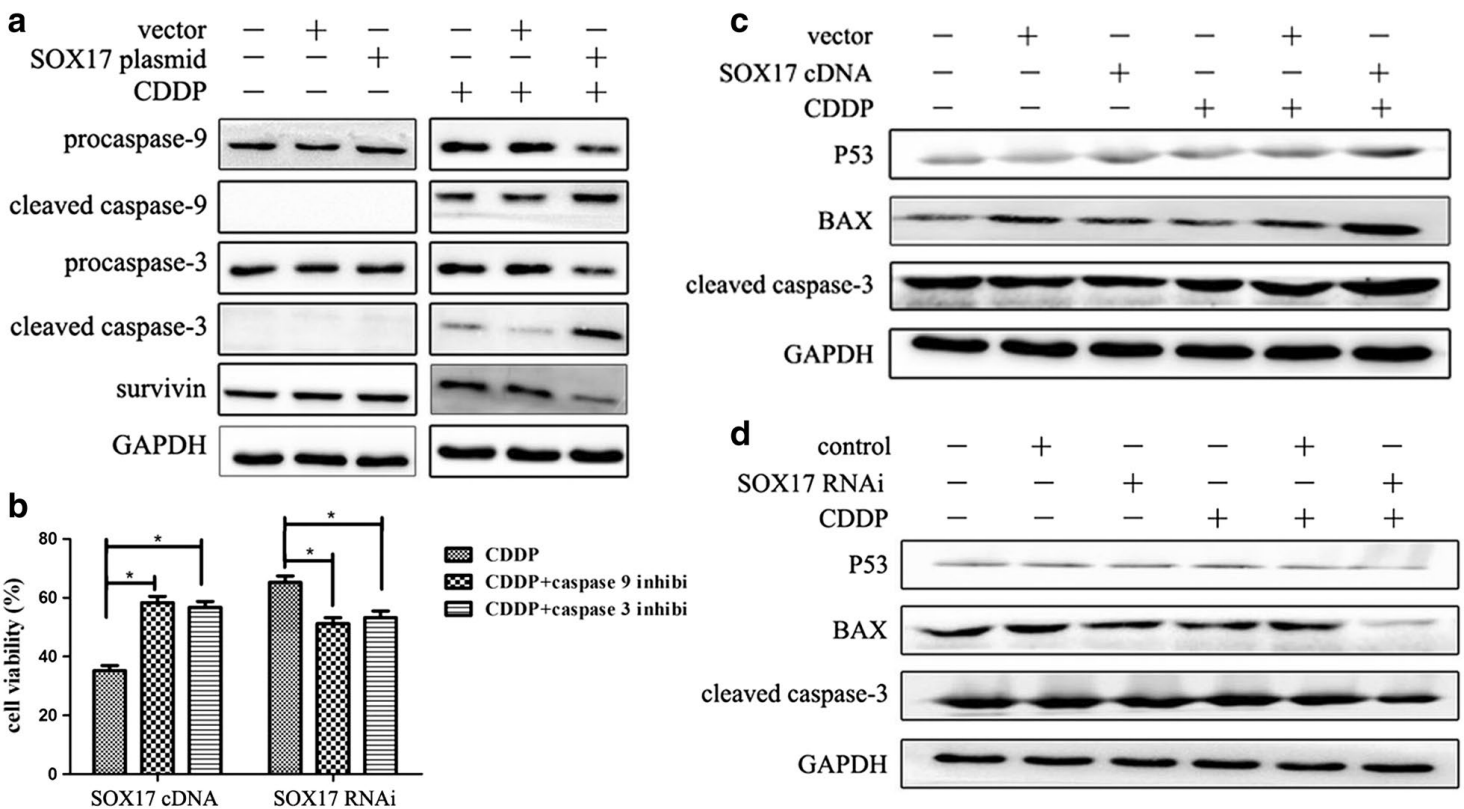
SOX17 induces mitochondrial apoptosis in EC cells after CDDP treatment. **a** Western blot experiments were performed to detect the levels of proteins. SOX17 overexpression increased the expression of cleaved caspase-3 and caspase-9. **b** CDDP regulated caspase-dependent apoptosis in HEC-1B cells. Cells were treated with 50 μM caspase-9 or caspse-3 inhibitor for 2 h and MTT analyses were performed after 48 h. **c**, **d** Down-regulated the p53-Bax-caspase-3 pathway in HEC-1B cells in vitro. Western blot analyses were performed to detect the protein levels of p53, Bax and cleaved caspase-3 (*P < 0.05)

SOX17 overexpression increased the levels of apoptosis in EC cells. We next investigated the role of SOX17 in regulating cell apoptosis after treatment with CDDP (20 μg/ml) for 48 h. Cleaved caspase-9, the marker of mitochondrial apoptosis activation, increased with higher SOX17 expression. As a result, cleaved caspase-3 (cc-3), the executor of apoptosis, increased, whereas the level of survivin, an inhibitor of apoptosis, decreased (Fig. 4a). Similarly, pretreatment with inhibitors of caspase-9 (ZLEHD-FMK) or caspase-3 (Z-DEVD-FMK) inhibited CDDP-induced activation of mitochondrial apoptosis in HEC-1B cells (Fig. 4b).

### SOX17 increased the p53-Bax-caspase-3 pathway in vitro

To further evaluate the involvement of SOX17 in the mitochondrial apoptotic events after CDDP treatment, the levels of the key proteins of the p53 pathway were analyzed. After SOX17 knockdown or overexpression, HEC-1B cells were treated with CDDP (20 μg/ml) for 48 h, and then assayed for the expressions of total proteins. SOX17 overexpression increased the level of p53 protein, whereas a reduced expression of SOX17 resulted in decreased p53. We also found that the downstream product of p53, Bax pro-apoptosis protein and cc-3, the executor of apoptosis, increased with SOX17 overexpression (Fig. 4c) and decreased with SOX17 under-expression (Fig. 4d). These results suggested that SOX17 altered the levels of pro- and anti-apoptotic proteins of the Bcl-2 family and influenced the CDDP sensitivity through the p53-Bax-caspase-3 apoptotic pathway.

### SOX17 increased the sensitivity of tumor cells to CDDP in vivo

**Fig. 5:**
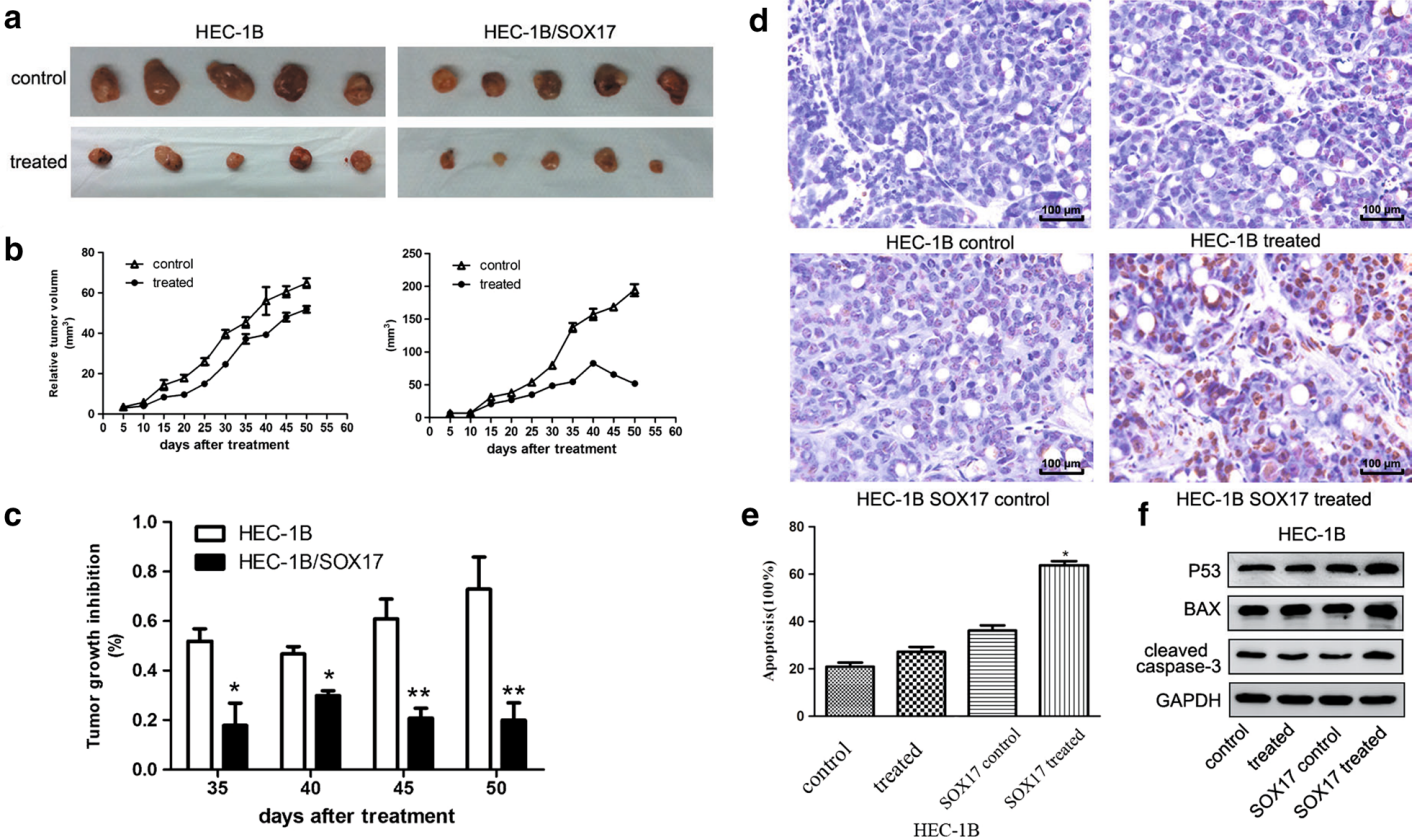
SOX17 influenced the sensitivity of tumor cells to cisplatin in vivo. The mice were inoculated with HEC-1B or HEC-1B-SOX17 cells and given saline (control group) or cisplatin (treatment group). **a** The tumors of HEC-1B-SOX17-treated cells were smaller than those of the HEC-1B-treated group. **b** Tumor growth curves of HEC-1B-treated cells (*left*) and of HEC-1B-SOX17-treated cells (*right*). The data are presented as the mean ± SD of five tumors per group. **c** The comparison of the cisplatin-induced growth inhibition of tumors from the two groups (*P < 0.05, **P < 0.001). **d**, **e** A higher apoptosis rate was exhibited in HEC-1B-SOX17-treated endometrial cancer cells than that in HEC-1B-treated cells as determined by TUNEL analysis (*P < 0.05). **f** Western blot analysis showing an elevated expression of p53, Bax and cleaved caspase-3 proteins in HEC-1B-SOX17-treated cells

To further investigate the effects of SOX17 on the resistance of EC cells to CDDP, an in vivo chemosensitivity experiment was performed by the subcutaneous transplantation of transduced cells into BALB/c nude mice. Cells were suspended at a density of 10^7^ cells/ml and 100 μl was injected into the flank of each nude mouse (n = 5). Nine days post-injection, CDDP (10 mg/kg) was intraperitoneally administered every 2 days for 8 days. Three days after the first treatment with CDDP, we measured the size of the growing tumors every 5 days for 50 days using the formula (W^*2*^ × *L*) (Fig. 5b, c). The mice were then euthanized and the tumors were excised from the nude mice. The tumor sizes of the SOX17 group were significantly smaller than those of the control group (Fig. 5a).

In addition, tumor growth curves indicated that after 10 days treatment, the tumors of the SOX17 group were smaller than those of the control. TUNEL assays on the tumor samples confirmed the hypothesis that the inhibition of tumor growth in the SOX17 group was due to increased cell apoptosis (Fig. 5d, e). To further explore the mechanism underlying this phenomenon, western blot analysis was performed to measure the expression levels of p53, Bax, and cc-3 (Fig. 5f). The tumors from the SOX17 group contained a significantly higher level of these proteins compared with those of the control group.

## Discussion

The mainstay of the initial treatment for EC is surgery. Chemotherapy is appropriate for patients with a high risk of extrauterine spread, including advanced stage carcinoma with deep myometrial invasion and poor differentiation [2]. Cisplatin-based two-drug combination chemotherapy has been the standard of care for patients with clinical stage II/III EC [2]. Cisplatin resistance is the most common cause of chemotherapy failure in EC. SOX17, a member of the SOX F family [6], is a transcription factor strongly involved in tumorigenesis; however, the role of SOX17 in the resistance of EC cells to CDDP remains poorly understood.

We discovered in this study that SOX17 expression was reduced in chemo-resistant EC tissues. To examine the molecular mechanisms underlying CDDP resistance in EC, SOX17 levels were assayed in a total of 42 patients after cisplatin-based combination chemotherapy. Results showed that SOX17 expression in chemoresistant patients was markedly decreased compared with that of chemosensitive patients. Based on these data, we hypothesize that SOX17 may be involved in the mechanism of cancer resistance to cisplatin chemotherapy.

Cisplatin is the first-line chemotherapy drug, of which the most prominent mode of anticancer action involves the generation of DNA lesions followed by the activation of the DNA damage response and the induction of mitochondrial apoptosis [18]. Mitochondrial-mediated apoptosis is initiated by several intracellular stimuli. The caspase initiator of the mitochondrial pathway results in Bax- or Bak-mediated cytoplasmic release of mitochondrial cytochrome C [19, 20]. The effector caspase, caspase-3 exerts the final execution of apoptosis through degradation of vital proteins involved in cell structure, signaling, cell cycle control and DNA repair [21].

The induction of apoptosis is one of the main purposes of anti-cancer drugs, and therefore resistance to apoptosis is suggested as a possible mechanism leading to the chemical unresponsiveness of cancer cells [22]. We first investigated the role of SOX17 in apoptosis of EC cells treated with CDDP. With the stably transfected cell lines, SOX17 overexpression increased the level of apoptosis. However, the molecular mechanism of SOX17 in regulating apoptosis of cancer cells after CDDP treatment is not fully understood.

Protein p53 is a tumor suppressor that triggers cell apoptosis [23]. Based on results from recent studies, it is proposed that, under pro-apoptotic conditions, p53 can play a dual role in apoptosis in different cellular compartments. Within the nucleus, p53 acts as a transcriptional activator and induces target gene expression through its interaction with the basic transcriptional machinery components leading to transcription-dependent apoptosis. In the mitochondria, p53 interacts with Bcl-2 and Bcl-X_L_, thereby triggering transcription-independent apoptosis. Here, p53 can lead to the release of the effectors Bak/Bax and elicit cytochrome C release [24].

In the present study, we found that SOX17 increased the expression of cleaved caspase-9 and caspase-3 after CDDP treatment. To explore the mechanisms underlying this phenomenon, we analyzed the expression levels of the proteins in the p53 pathway involved in apoptosis. The results indicated that SOX17 increased the expression levels of the proteins in this pathway. To confirm this effect, we pretreated cells with inhibitors for caspas-9 and caspas-3. As expected, the efficacy of SOX17 in CDDP-induced apoptosis was suppressed. These data suggested that SOX17 affected CDDP chemosensitivity through p53-triggered mitochondrial-mediated apoptosis.

The in vitro, data also suggested that the knockdown of SOX17 markedly decreased the CDDP sensitivity of tumor cells by up-regulating the expression of p53, Bax and cc-3 proteins.

In breast cancer tissue with mutant p53, the transcription factor SOX17 is hypermethylated [16]. A more recent study showed that SOX30, a member of the SOX family containing a highly conserved HMG DNA-binding domain, bound directly to the p53 promoter region, and the SOX30 HMG-box was required for stimulating p53 promoter activity [25]. SOX17, a member of the SOX transcription factor superfamily, has the HMG box, and can possibly directly or indirectly bind to the p53 promoter region. How SOX17 up-regulates p53 levels remains to be determined.

Point mutations in p53 are found in 90 % of cases of type II endometrial cancer, but in only 10–20 % of cases of grade 3 type I EC[26]. p53 gene mutations occurred only at sites with positive p53 protein expression in endometrioid adenocarcinoma, which were poorly differentiated regions of cancer tissue [27]. In this study, we selected only the p53 mutant cell line HEC-1B to explore the function of SOX17 in chemoresistance to CDDP. Whether SOX17 influences the chemosensitivity of p53 null-EC cells to CDDP, or affects the chemosensitivity of p53 mutant non-endometrioid cells, needs further study.

## Conclusion

Our results indicate that SOX17 is involved in the p53-mediated apoptosis pathway, and increases the sensitivity of HEC-1B cells to cisplatin. A precise elucidation of the mechanism of SOX17 on this signaling is necessary for understanding CDDP chemoresistance and apoptosis.

## Methods

### Cells and procurement of patient samples

The protocols used in the study were approved by the Hospital’s Protection of Human Subjects Committee. Samples were acquired with written informed consent from the Shanghai First People’s Hospital Affiliated to Shanghai Jiaotong University.

Paraffin-embedded tissues of 42 EC patients were included in the study, and all the patients received cisplatin-based combination chemotherapy between 12/2013 and 08/2014. The average age of the patients with EC was 53.9 ± 3.8 years (mean ± SD; median, 55 years; range, 41–63 years). The difference between the trial and the control was not significant (*P* = 0.317). The stages (II–III) and histological grades (G2–G3) of these tumors were established according to the criteria of the International Federation of Gynecology and Obstetrics (FIGO) surgical staging system (2009) [28]. None of the patients had undergone hormone therapy or radiotherapy before surgery.

A total of 29 EC patients were included in the study, 17 being cisplatin-sensitive and 12 cisplatin-resistant. Blood specimens of these patients were acquired, and all the patients received cisplatin-based combination chemotherapy between 12/2013 and 08/2014 (median age 54.3 years, range 43–58 years).

Human EC cell lines Ishikawa, HEC-1B, AN3CA and SPEC-2 were obtained from the China Center for Type Culture Collection (CCTCC) and maintained as recommended by the American Type Culture Collection (ATCC, Manassas, VA).

### Immunohistochemistry (IHC) and assessments

Immunohistochemical staining was performed using the 2-Step Plus Poly-HRP Anti IgG Detection System (ZSGB-Bio, Beijing, China) according to the manufacturer’s recommendations. Mouse polyclonal anti-SOX17 (diluted 1:100–10 μg/ml; Abcam, Cambridge, UK) was used as the primary antibody. Scoring was performed by two independent pathologists who had not seen the clinical and pathological data. Protein staining was evaluated as described [29].

### RNA extraction and analysis

The extraction of total RNA and reverse transcription (RT) reactions were performed according to the manufacturer’s protocol. Real-time PCR was performed using a standard protocol from the SYBR Green PCR kit (Toyobo, Osaka, Japan). Glyceraldehyde-3-phosphate dehydrogenase (GAPDH) mRNA was used as a reference for mRNAs. DCt values were normalized to GAPDH mRNA levels. The 2^−△△Ct^ method was used to determine the relative gene expression levels. The primer pairs used were human SOX17 forward: 5′-ATCCTCAGACTCCTGGGTTT-3′, reverse: 5′-ACTGTTCAAGTGGCAGACAAA-3′. GAPDH forward: 5′-GGCTCCCTTGGGTATATGGT-3′, reverse: 5′-TTGATTTTGGAGGGATCTCG-3′.

### Western blot analysis

Western blot analysis to assess protein expression was performed as previously described [30]. Primary antibodies included mouse anti-SOX17 (Abcam, Cambridge, UK) and mouse anti-GAPDH (Sigma, ST Louis MO, USA). All the other antibodies were purchased from CST (Boston, USA).

### Cell culture and transfection

Transient transfection of HEC-1B cells was performed with lipofectamine 2000 following the manufacturer’s recommendations. Tumor cell clone overexpressing SOX17 was successfully established as previously described [31]. Cells were cultured in 10 % Dulbecco’s Modified Eagle’s Medium (DMEM) containing 10 % fetal bovine serum (Wisent, Quebec, Canada) at 37 °C with 5 % CO_2_.

### Analysis of cell apoptosis

The percentage of cells undergoing apoptosis was evaluated using simultaneous Annexin V-FITC and propidium iodide (PI) staining. Cells were treated with CDDP (20 μg/ml) for 48 h. The apoptotic cells were analyzed by flow cytometry on a FACScan (Beckton-Dickson, New Jersey, USA).

### Cell viability assay

Cells were plated for 24 h, then treated with CDDP for an additional 48 h. The number of viable cells was determined by using the 3-(4,5-dimethylthiazol-2yl)-2,5-diphenyltetrazolium bromide (MTT) assay as described [32].

### Flow cytometry

Cells were incubated with CDDP for 48 h. The subsequent steps were performed according to the manufacturer’s protocol. DNA content was measured on a FACSCalibur-HTS flow cytometer (BD Biosciences, New Jersey, USA).

### RNAi and overexpression

We designed an oligonucleotide as the target genes of SOX17 mRNA in accordance with the law of multiple biological information (GeneBank number NM 022454). The RNA interference target sequence: 5′-GGTATATTACTGCAACTAT-3′. A BLAST search confirmed the non-silencing siRNA had no matches with the complete human genome (http://www.ncbi.nlm.nih.gov). The sequence of NC target gene is 5′-TTCTCCGAACGTGTCACGT-3′. ShRNA was cloned into pCDNA3.1 vector. The positive clones were identified by PCR and sequenced. SOX17cDNA plasmid was purchased from Origene (Cat no. RC220888, USA).

### Terminal deoxynucleotidyl transferase-mediated dUTP nick end-labeling (TUNEL) assay

All tumors were collected by sacrificing the mice 50 days after treatment. In situ Death Detection Kits (Roche, Mannheim, Germany) were used to detect apoptotic cells by specific staining. TUNEL assays for determining cell apoptosis were performed according to the manufacturer’s protocol. The apoptotic rate of cancer cells was calculated as apoptotic cells/total cells ×100 %.

### Statistical analysis

All experiments were repeated in triplicate. Data were expressed as the mean ± SD. The statistical significance between two groups was determined by Student’s *t* test. The association between SOX17 expression and clinicopathological parameters was examined by the Chi square test. *P* < 0.05 were considered statistically significant.

## Additional file

10.1186/s12935-016-0304-7 Clinical characteristic data. **Table S2**: IHC data.
