# Supplementary material for: SOX17 increases the cisplatin sensitivity of an endometrial cancer cell line
Source: Cancer Cell Int. 2016 Apr 8;16:29. doi: 10.1186/s12935-016-0304-7 (PMC4826500; doi:10.1186/s12935-016-0304-7)
Supplement: Supplementary file 1 — 10.1186/s12935-016-0304-7 Clinical characteristic data. Table S2: IHC data. [file 12935_2016_304_MOESM1_ESM.docx]

**Table S1.** **Clinical Features of the patients** ^a^

| characteristic | Sensitivity n(%) | Resistance  n(%) | Total  n(%) | *P* |
| --- | --- | --- | --- | --- |
| age |  |  |  | 0.317 |
| <50 | 16 (76.19) | 13 (61.90) | 29 (69.05) |  |
| ≥50 | 5 (23.81) | 8 (38.10) | 13 (30.95) |  |
| grade |  |  |  | 0.725 |
| П | 6 (28.57) | 5 (23.81) | 11 (26.19) |  |
| Ш | 15 (71.43) | 16 (76.19) | 31 (73.81) |  |
| stage |  |  |  | 0.214 |
| II | 5 (23.81) | 2 (9.52) | 7 (16.67) |  |
| III | 16 (76.19) | 19 (90.48) | 35 (83.33) |  |

Abbreviations: a Values are given as median, or number (percentage), unless indicated otherwise.

**Table S2. SOX17 expression in endometrial cancer tissues** ^a^

| SOX17expression | Chemosensitivity group (case) | Chemoresistant group (case) |
| --- | --- | --- |
| - | 1 | 5 |
| 1+ | 2 | 4 |
| 2+ | 3 | 5 |
| 3+ | 7 | 3 |
| 4+ | 8 | 4 |
| total | 21 | 21 |

Abbreviations: a Values are given as number, unless indicated otherwise.
